# Fucoidan Inhibits the Progression of Hepatocellular Carcinoma *via* Causing lncRNA LINC00261 Overexpression

**DOI:** 10.3389/fonc.2021.653902

**Published:** 2021-04-13

**Authors:** Danhui Ma, Jiayi Wei, Sinuo Chen, Heming Wang, Liuxin Ning, Shi-Hua Luo, Chieh-Lun Liu, Guangqi Song, Qunyan Yao

**Affiliations:** ^1^ Department of Gastroenterology and Hepatology, Zhongshan Hospital of Fudan University, Shanghai, China; ^2^ Shanghai Institute of Liver Diseases, Shanghai, China; ^3^ Department of Traumatology, Rui Jin Hospital, School of Medicine, Shanghai Jiao Tong University, Shanghai, China; ^4^ Department of Clinical Research and Development, Hi-Q Marine Biotech International Ltd., Taipei, Taiwan

**Keywords:** fucoidan, hepatocellular carcinoma, long non-coding RNA, high-throughput sequencing, LINC00261

## Abstract

Hepatocellular carcinoma (HCC) as a main type of primary liver cancers has become one of the most deadly tumors because of its high morbidity and poor prognosis. Fucoidan is a family of natural, heparin-like sulfated polysaccharides extracted from brown algae. It is not only a widely used dietary supplement, but also participates in many biological activities, such as anti-oxidation, anti-inflammation and anti-tumor. However, the mechanism of fucoidan induced inhibition of HCC is elusive. In our study, we demonstrated that fucoidan contributes to inhibiting cell proliferation *in vivo* and *in vitro*, restraining cell motility and invasion and inducing cell cycle arrest and apoptosis. According to High-Throughput sequencing of long-non-coding RNA (lncRNA) in MHCC-97H cells treated with 0.5 mg/mL fucoidan, we found that 56 and 49 lncRNAs were correspondingly up- and down-regulated. LINC00261, which was related to the progression of tumor, was highly expressed in fucoidan treated MHCC-97H cells. Moreover, knocking down LINC00261 promoted cell proliferation by promoting the expression level of miR-522-3p, which further decreased the expression level of downstream SFRP2. Taken together, our results verified that fucoidan effectively inhibits the progression of HCC *via* causing lncRNA LINC00261 overexpression.

## Introduction

As one of the most common types of cancer, hepatocellular carcinoma (HCC) is currently reported to be the third leading cause of cancer-related death worldwide (1) and accounts for 75% of all primary liver cancers (2). Epidemiology showed that the incidence rate of liver cancer has increased by nearly 4 times over the past 40 years (3). Surgical interventions are usually the main treatment for early HCC. However, the five-year recurrence rate after surgery reaches up to 50%-70% and only 15% of these cases could be resected repeatedly, which greatly limits the therapeutic effect of surgical resection (4). Interventional therapy such as transcatheter arterial chemoembolization (TACE) is recommended as the standard treatment for patients with unresectable liver cancer. Although TACE prolongs the life of patients with advanced liver cancer to a certain extent, some patients develop TACE refractoriness after two cycles of treatment resulting in insufficient treatment effect and the mortality related to side effects of TACE reaches 0.5% (5, 6). Traditional cytotoxic drugs are limited in clinical application due to their high toxicity and side effects. While first-line recommended molecular targeted drugs, such as sorafenib and lenvatinib, are superior to traditional chemotherapy drugs in terms of safety and efficacy, which could only prolong the survival time of patients for several months due to the serious decline of liver function in patients with advanced HCC (7). The cell programmed death protein-1 (PD-1) inhibitors also showed definite efficacy only in 15%-20% of patients with advanced HCC (8). HCC has become one of the most malignant tumors with extremely difficulty to cure due to its high recurrence rate and strong drug resistance. Therefore, it is urgent to reveal the mechanism of HCC occurrence and development, develop new targets and effective targeted molecules.

Fucoidan is a family of natural, heparin-like sulfated polysaccharides extracted from brown algae. Its structure is highly dependent on the species of algae, but has common characteristics of the backbone of sulfated fucoidan (9). In the past few years, attention was paid to the research of fucoidan and large numbers of products containing fucoidan extract were developed. In 2003, a drug named “Haikun Shenxi Capsule” was approved for the treatment of chronic renal failure in China (10). In the United States and Europe, the use of fucoidan in supplements and cosmetics was also ratified by regulatory authorities (11). Till now, a series of studies have reported its effects on anti-oxidation, anti-coagulation, anti-thrombosis, immune regulation, anti-virus (12–16). Recent research demonstrated that fucoidan have certain therapeutic value for fatty liver and liver fibrosis in zebrafish (17). Fucoidan was also confirmed to have an anti-tumor effect, and as a natural product, it is considered to be safer than other classic chemotherapy drugs. This polymer could reduce the migration and metastasis ability of cancer cells (18), but also play an anti-tumor effect through arresting cell cycle, inducing apoptosis (19, 20), enhancing cancer immunity (21, 22) or inhibiting tumor angiogenesis (23). Since the mechanism of fucoidan in the treatment of HCC was not fully confirmed, our study will mainly explore the molecular mechanism of fucoidan in the occurrence, development and metastasis of HCC.

According to statistics, only about 2% of all the transcripted mammalian genomes are involved in protein coding (24). Of all the non-coding RNAs, those with length greater than 200 nucleotides were further identified as lncRNAs (25). At the very beginning, lncRNAs were regarded as the “garbage” of genome transcription, which was the by-product of RNA polymerase II transcription and had no biological function. With the development of High-Throughput sequencing technology, our understanding of lncRNAs was gradually improved. Through the progression of various studies in recent years, lncRNAs were observed to participate in complex biological activities. They serve as RNA scaffolds (26, 27), RNA guides (28), RNA decoys (29) or molecular sponges (30, 31) to regulate cell growth, differentiation, and establish cell identity, which are usually uncontrolled in cancer. Various studies already revealed that the aberrant expression and mutations of lncRNA are closely related to tumor occurrence and metastasis (32–35). H19 (36–38), HOTAIR (39–41), MALAT1 (42–44) and some other well-studied lncRNAs were proved to be able to influence the development of liver cancer through a variety of cellular regulatory mechanisms, such as regulating tumor suppressor genes (36), affecting cell cycle and apoptosis factors (45, 46), impacting the function of downstream microRNAs (miRNA) (30) and interrupting signaling pathways (35). Moreover, lncRNAs are also considered to be involved in regulating the characteristics of cancer stem cells (47) and may be related to the generation of drug resistance in some tumors (48, 49). Therefore, it is very promising to identify lncRNA functions, figure out their relationship with HCC and develop new strategies for diagnosis and trageted therapy based on lncRNAs.

Compared with small molecule chemical drugs, natural products have higher therapeutic safety. They also could affect the expression level of lncRNAs, which is crucial for tumor progression (50–53). In this research, we confirmed that fucoidan could effectively inhibit the progression of HCC. High-Throughput sequencing was used to detect and obtain the expression profile of lncRNAs in MHCC-97H cells treated with fucoidan. According to the analysis results, differentially expressed lncRNAs after fucoidan treatment, which are related to the occurrence and development of HCC, were selected to verify the effect of fucoidan. In this study, we demonstrated that fucoidan inhibits the proliferation and invasion of HCC cells by up-regulating LINC00261. Overexpression of LINC00261 regulates downstream miR-522-3p to play an anti-tumor role, which may provide potential new molecules and new targets for clinical treatment of HCC. The results demonstrated that LINC00261 inhibits proliferation and invasion of HCC cells by regulating miR-522-3p, which provided potential new molecules and new targets for clinical treatment of liver cancer.

## Materials and Methods

### Cell Culture

The MHCC-97H and Hep3B cell lines were obtained from Zhongshan Hospital of Fudan University. MHCC-97H and Hep3B cells were cultured in complete DMEM supplemented with 10% fetal bovine serum, 1% penicillin and streptomycin. The cell culture environment was 37 °C with 5% CO2.

### Reagents and Antibodies

Fucoidan powder was acquired from Hi-Q Marine Biotech International Ltd. Saline was used as solvent for fucoidan solutions. The storage concentration was 5 mg/mL, and then further diluted to the final required concentration (0.25 mg/mL and 0.5 mg/mL). The powder and solution are sealed and stored at 4 °C. Antibody against SFRP2 was purchased from Proteintech (12189-1-AP).

### Cell Morphological Examination

1×10^5^ cells per well were seeded on the 12-well plate overnight. The adherent cells were then treated with saline, 0.25 mg/mL and 0.5 mg/mL fucoidan for 48 h. The cell morphology was recorded under microscope (Olympus, IX73) at 48 h after fucoidan treatment. All the pictures were taken under 20 times magnification. Each group had three independent duplications.

### Cell Proliferation

1×10^5^ cells per well were seeded on 6-well plate overnight. The adherent cells were treated with 0.25 mg/mL and 0.5 mg/mL fucoidan for 48 h. Cells are digested and resuspended after 48 h and counted by blood counting chamber and presented as the mean ± standard deviation (SD) from three independent duplications.

### Cell Viability Assay (CCK-8 Assay)

We used the WST-8-based detection method to analyze the cell viability according to the instructions of a CCK-8 kit (Yeasen). 2000 cells were seeded to the 96-well plate overnight. The adherent cells were treated with 0.25 mg/mL and 0.5 mg/mL fucoidan for the indicated times (0, 24, 48 and 72 h). 10 μL CCK-8 reagent and 90 μL medium were added per well and incubated for 2 h. After incubation, a multifunctional reader (MD FlexStation3) was used to detect the absorbance of the cells at 450 nm. There were three repetitions in each group and values were presented as the mean ± standard deviation (SD).

### Clone Formation Assay

2500 cells were seeded on 6-well plates overnight and were then treated with 0.5 mg/mL fucoidan for 7-10 days according to the cell condition. The cells were then fixed with 4% paraformaldehyde for 15 min. After that, the fixative was washed out, and 0.1 g/mL crystal violet was added for 15 min for staining. The clones were recorded by camera and was counted by ImageJ. Each group had three independent repetitions.

### Wound Healing Assay

6×10^5^ cells were seeded into the 6-well plate. The cells were cultured in complete DMEM for 12 h to 90%-95% density, sterile pipette tip was used to draw a line vertically in the middle of each well. Then, cell debris was removed with preheated PBS twice, and the cells were treated with 0.25 mg/mL and 0.5 mg/mL fucoidan in FBS-free DMEM. Images were collected 0 h and 48 h later to measure the distance of wound. The wound closure rate is equal to the distance of the wound at 48 h divided by the distance of the wound at 0 h. There were three independent repetitions in each group and values were presented as the mean ± standard deviation (SD).

### Cell Invasion Assay

Transwell assay was carried out by using 8 μm transwell chamber according to the manual (BD Science). 1×10^5^ cells per well were digested and washed with serum-free DMEM for three times before seeding into the upper chamber. Serum-free DMEM was used to dissolve fucoidan to 0.5 mg/mL and the cells were resuspended at a volume of 100 μL per well and planted into the upper chamber, while 600 μL DMEM supplemented with 20% FBS and 0.5 mg/mL fucoidan was added into the lower chamber. After 48 h, the films were removed, washed with PBS twice and fixed with 4% paraformaldehyde for 15 min. 0.1% crystal violet was used to stain the film for 15 min and then the upper side of the film was carefully wiped with cotton swabs. The cells were recorded with microscope and the total area of cells was counted by ImageJ and values were presented as the mean ± standard deviation (SD). Each group had three independent repetitions.

### Cell Cycle Assay

After seeding 6-well plates with 8×10^5^ cells per well, the cells were cultured in medium containing 0.25 mg/mL, 0.5 mg/mL fucoidan for 48 h. The cell cycle was detected by a cell cycle and apoptosis kit (Beyotime Biotechnology) and flow cytometry according to standard instructions.

### Cell Apoptosis Assay

Cell apoptosis was analyzed by flow cytometry as per the FITC-conjugated Annexin V/PI method (Annexin V-FITC/PI apoptosis kit, Beyotime Biotechnology). 2×10^5^ cells were seeded into the 6-well plates. After 48 h treatment of 0.25 mg/mL and 0.5 mg/mL fucoidan, the adherent and suspended cells in each well were collected and resuspended in 195 μL binding buffer, following which Annexin V-FITC (5 μL) and PI (10 μL) were added to each sample and mixed. Cells were incubated for 15 min in the dark at 4 °C and then analyzed by flow cytometry. Each group had three independent repetitions and values were presented as the mean ± standard deviation (SD).

### RNA Extraction and qRT-PCR

Total RNA was extracted by TRIzol (Invitrogen) according to the manual. Then the RNA was reverse-transcribed to cDNA using the Hifair^®^ III 1st Strand cDNA Synthesis SuperMix kit (Yeasen) following the manufacturer’s instructions. The concentration and purity of each sample was tested by DS-11 Spectrophotometer (DeNovix). Equivalent amounts of cDNA were used for real-time PCR in a 20 μL reaction mixture using the Hieff^®^ qPCR SYBR^®^ Green Master Mix kit (Yeasen). Primers for qRT-PCR were as shown in **Supplementary Table S3**. The internal reference gene for lncRNA was GAPDH. QuantStudio5 (Applied Biosystems™) was used to perform the real-time fluorescent quantitative PCR, while ProFlex PCR system (Applied Biosystems™) was used to perform reverse-transcription. Relative levels in each sample were calculated based on their threshold cycle (Ct) values, 2^-ΔΔCt^ method was used. Each sample had three independent repetitions.

### siRNA Interference

The siRNA for LINC00261 knockdown was customized from GenePharma. Before transfection, cells were plated in 60 mm dish and transfected with si-LINC00261 at the 60%-80% confluence. The ribo FECT^TM^ CP Transfection kit (RiboBio) was used to perform the transfection according to the manufacturer’s protocol. Every siRNA had a scrambled siRNA as negative control. The cells were incubated at 37 °C in a 5% CO2 incubator for 48 h, then the LINC00261 expression was examined by qRT-PCR.

### High-Throughput Sequencing

To verify the change of lncRNA and mRNA expression after fucoidan treatment, High-Throughput sequencing was performed by GENEWIZ^®^. The cells were treated with saline and 0.5 mg/mL fucoidan for 48 h in T25 flasks. Cells were then digested and transferred into 1.5 mL tubes, the number of cells per tube is around 3×10^5^. Total RNA was extracted by TRIzol (Invitrogen) according to the manual and was frozen to -80 °C. Ribosomal deletion RNA was used to build the sequencing library, thus the Illumina NovaSeq machine (Illumina) using a 2×150 paired-end (PE) configuration was used to perform the sequencing. Data with adapters and QCs less than 20 was removed using cutadapt (v1.9.1) to get the final clean record. Next, we used Hisat2 (v2.0.1) to analyze and map the clean data to the reference human genome. The count of each transcript was obtained by using rsem (v1.2.15) after quantification and annotation. Finally, the transcripts were standardized and analyzed for differential expression using DESeq2. The sequencing data was submitted to the Sequence Read Archive (SRA) data set with registration number PRJNA (PRJNA690771).

### Differential Expression Analysis

Differential expression analysis used the DESeq2 or EdgeR Bioconductor package, a model based on the negative binomial distribution. The estimates of dispersion and logarithmic fold changes incorporate data-driven prior distributions, Padj of genes were setted <0.05 to detect differential expressed ones.

### GO and KEGG Enrichment Analysis

GOSeq(v1.34.1) was used to identify Gene Ontology (GO) terms that annotate a list of enriched genes with a significant Padj less than 0.05. KEGG (Kyoto Encyclopedia of Genes and Genomes) is a collection of databases dealing with genomes, biological pathways, diseases, drugs, and chemical substances (http://en.wikipedia.org/wiki/KEGG). We used scripts in house to enrich significant differential expression gene in KEGG pathways.

### Xenograft Tumor Model

All animal investigation in our study was conformed to the guidelines of Animal Care and Use Committee, Zhongshan Hospital of Fudan University. Balb/c nude mice were purchased from Vital River Laboratory Animal Technology Co., Ltd. 1×10^7^ MHCC-97H cells were subcutaneously injected into 4 weeks old female mice. After two weeks, mice bearing tumors were randomly divided into two groups: the Ctrl group (saline) and the Fuc group (15 mg/kg per day). Each group consisted of five mice, which were orally fed for three weeks. Body weights of mice were measured every week. Tumor volume was measured by using the formula *V* = (*a* × *b* ^2^)/2 (*V* is volume, *a* is the length of the tumor, *b* is the width of the tumor).

### Statistical Analysis

Statistical analysis was performed using GraphPad 8.4.3. The analysis was carried out using Student’s t test (N.S. means not significant, * means P < 0.05, ** means P < 0.01, *** means P < 0.001, **** means P < 0.0001).

## Results

### Fucoidan Inhibits Proliferation of HCC *In Vitro* and *In Vivo*

**Figure 1:**
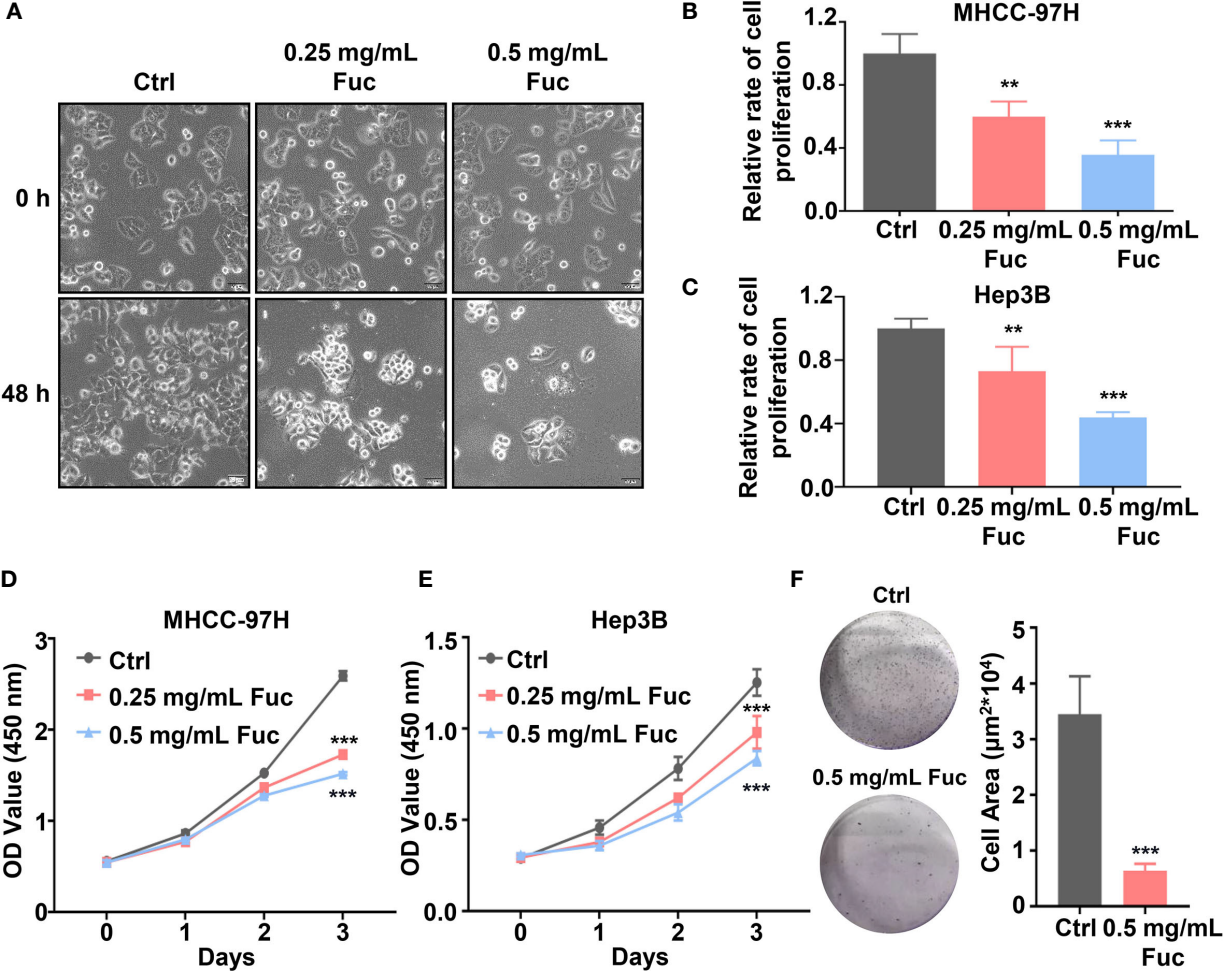
Fucoidan inhibits proliferation of hepatocellular carcinoma *in vitro*. **(A)** Morphology of MHCC-97H cells treated with saline (Ctrl), 0.25 and 0.5 mg/mL Fuc. **(B, C)** Relative cell proliferation rate of MHCC-97H **(B)** and Hep3B **(C)** cells after treatment with saline (Ctrl), 0.25 and 0.5 mg/mL Fuc. ** means P < 0.01, *** means P < 0.001. **(D, E)** Cell viability of MHCC-97H **(D)** and Hep3B **(E)** cells treated with saline (Ctrl), 0.25 and 0.5 mg/mL Fuc. *** means P < 0.001. **(F)** Colony formation results of MHCC-97H cells treated with saline (Ctrl) and 0.5 mg/mL Fuc. *** means P < 0.001.

The fucoidan we used contains a repetitive unit consisting of disaccharides including α-1,3-fucose and α-1,4-linked fucose with arms affixed to C2 position (54). To verify whether fucoidan could inhibit the proliferation of HCC, we treated MHCC-97H cells with saline (Ctrl), 0.25 and 0.5 mg/mL fucoidan (Fuc) for 48 h. Compared with the Ctrl group, fucoidan obviously restrained proliferation of MHCC-97H cells in a concentration-dependent manner and the morphological changes of cell apoptosis such as budding and vacuolation could be observed (**Figure 1A**). To further confirm this observation, we carried out cell proliferation experiment. Equal number of cells were treated with saline, 0.25 mg/mL and 0.5 mg/mL fucoidan respectively for 48 h. The results disclosed that the proliferation ability of the cells was slowed down after fucoidan treatment and the inhibitory effect was positively correlated with the dosages (**Figure 1B**). Similar results were obtained in the experiment repeated with Hep3B cells (**Figure 1C**). We additionally performed cell viability assay with both MHCC-97H and Hep3B cells respectively treated with saline, 0.25 mg/mL and 0.5 mg/mL fucoidan for 24 h, 48 h and 72 h. The results suggested that fucoidan suppressed cell viability and the inhibitory effect was manifested at 48 h (**Figures 1D, E**). The clone formation result further confirmed the inhibitory effect of fucoidan on HCC cell proliferation compared with the Ctrl group (**Figure 1F**).

**Figure 2:**
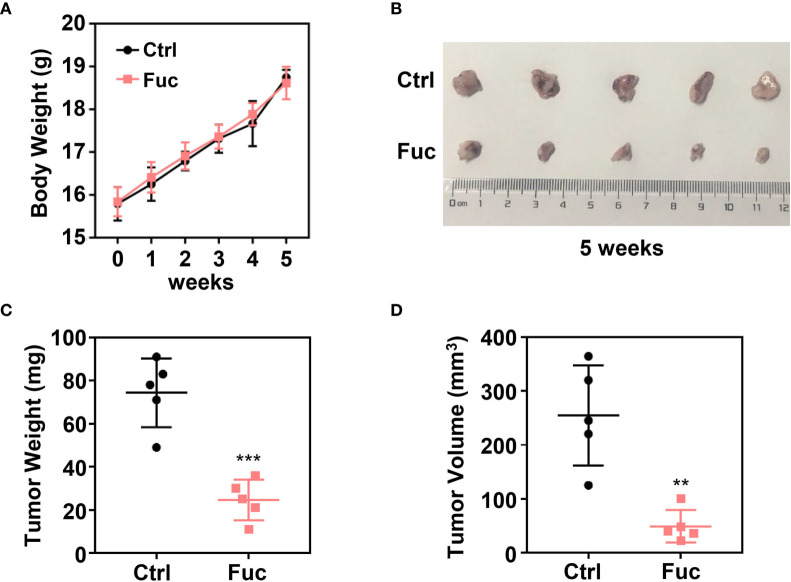
Fucoidan inhibits proliferation of hepatocellular carcinoma *in vivo*. **(A)** Body weight of the Ctrl group and Fuc group (15 mg/kg Fuc per day, dissolved in saline). **(B)** Xenograft tumor formation of the Ctrl group and Fuc group. **(C)** Tumor weight of the Ctrl group and Fuc group. *** means P < 0.001. **(D)** Tumor volume of the Ctrl group and Fuc group. ** means P < 0.01.

To determine whether fucoidan inhibit HCC cell proliferation *in vivo*, we performed xenograft tumor model. 1×10^7^ MHCC-97H cells were subcutaneously injected into 4 weeks old female Balb/c nude mice. After two weeks, 15 mg/kg Fuc were orally fed per day for three weeks. Body weights of mice were measured every week. Tumor weight and volume were recorded at the end of treatment. The results suggested that fucoidan not only is nearly no toxicity to mice (**Figure 2A**), but obviously decreased tumor weight and volume *in vivo* (**Figures 2B–D**).

### Fucoidan Inhibits Motility and Invasion of HCC Cells

**Figure 3:**
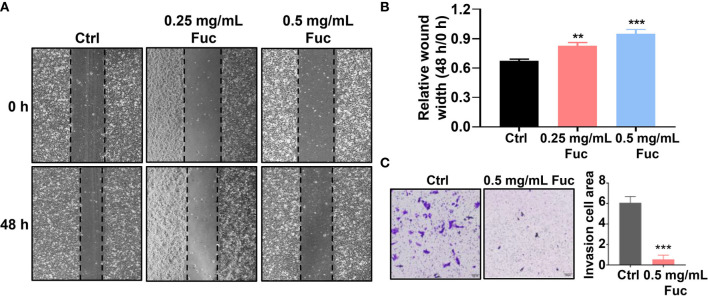
Fucoidan inhibits motility and invasion of hepatocellular carcinoma cells. **(A, B)** Wound healing results of MHCC-97H treated with saline (Ctrl), 0.25 and 0.5 mg/mL Fuc. ** means P < 0.01, *** means P < 0.001. **(C)** Transwell results of MHCC-97H treated with saline (Ctrl) and 0.5 mg/mL Fuc. *** means P < 0.001.

To determine whether fucoidan could affect cell motility in HCC, wound healing assay was performed. As shown in **Figure 3A**, the relative wound width of Ctrl group was significantly lower than that of fucoidan treatment group, while cells treated with 0.5 mg/mL fucoidan showed better performance than those treated with 0.25 mg/mL, suggesting that fucoidan has inhibitory effect on cell motility and the effect is also dose-dependent (**Figures 3A, B**). Transwell assay was further conducted to verify the effect of fucoidan on the invasion ability of HCC cells. The results revealed that the number of cells penetrating the membrane decreased in the fucoidan treatment group, which confirmed that fucoidan decreased the invasion ability of the HCC cells (**Figure 3C**). Based on the above results, we preliminarily confirmed that fucoidan could inhibit the proliferation, motility and invasion of HCC cells.

### Fucoidan Induces Apoptosis and Cell Cycle Arrest in HCC Cells

**Figure 4:**
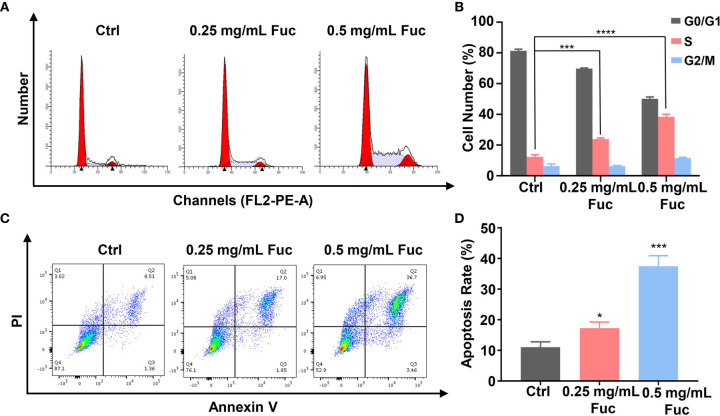
Fucoidan arrests cell cycle and promotes apoptosis of MHCC-97H cells. **(A, B)** Cell cycle distribution was measured by flow cytometry in MHCC-97H cells induced by saline (Ctrl), 0.25 and 0.5 mg/mL Fuc. *** means P < 0.001, **** means P < 0.0001. **(C, D)** Percentage of apoptotic MHCC-97H cells after treatment with saline (Ctrl), 0.25 and 0.5 mg/mL Fuc. Q1 represents death cells, Q2 represents the late apoptosis cells, Q3 represents the ealry apoptosis cells, Q4 represents the normal cells. The apoptosis rate equals to the rate of late apoptosis cells (Q2) plus the rate of early apoptosis cells (Q3). * means P < 0.05, *** means P < 0.001.

To gain further insight into the involvement of fucoidan in HCC development, we used flow cytometry to detect the cell cycle distribution and apoptosis rate of MHCC-97H cells treated with fucoidan. Similarly, we seeded the cells in medium with saline, 0.25 mg/mL and 0.5 mg/mL fucoidan for 48 h. As shown in **Figure 4A**, treatment of MHCC-97H cells with higher dosage of fucoidan increased the S phase distribution, which indicated that fucoidan could induce cell cycle arrest at S phase in a dose-dependent manner (**Figures 4A, B**). Meanwhile, higher apoptosis rate was observed in fucoidan treated group rather than that of the control group, which was positively correlated with the concentration of fucoidan. This result further prompts that fucoidan also promotes the apoptosis of HCC cells (**Figures 4C, D**). Therefore, our data demonstrated that fucoidan is able to arrest cell cycle and promote apoptosis of HCC cells.

### Fucoidan Contributes to the Alteration of lncRNAs Profiling in HCC

**Figure 5:**
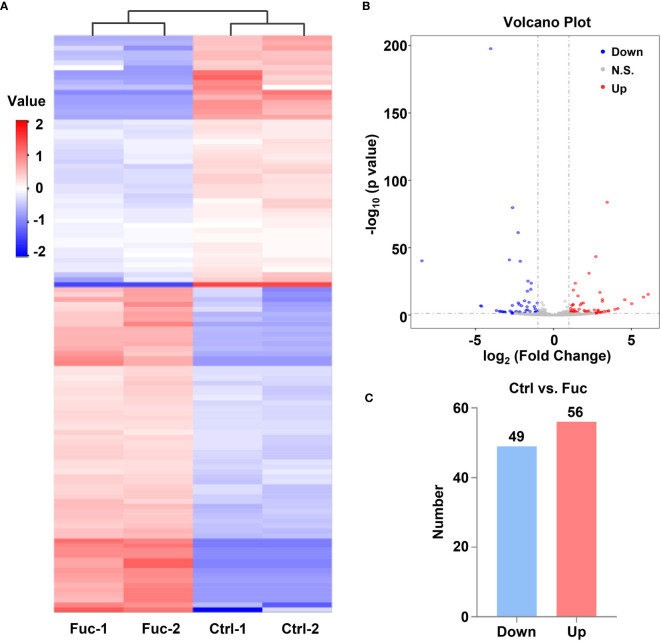
Fucoidan contributes to the alteration of lncRNAs profiling in MHCC-97H cells. **(A)** Heatmap shows clustering analysis of differentially expressed lncRNAs treated with saline (Ctrl) and 0.5 mg/mL Fuc. Each group had two duplicated experiments. **(B)** Volcano plots presents significantly different expression profiles of lncRNAs in MHCC-97H cells treated with saline and 0.5 mg/mL Fuc. Vertical lines referred to 2-fold changes in up-regulation and down-regulation. Horizontal line corresponds to p=0.05. Blue and red points represent to down- and up-regulation with statistical significance. **(C)** Numbers of significantly down- and up-regulated lncRNAs in MHCC-97H cells treated with 0.5 mg/mL Fuc.

**Figure 6:**
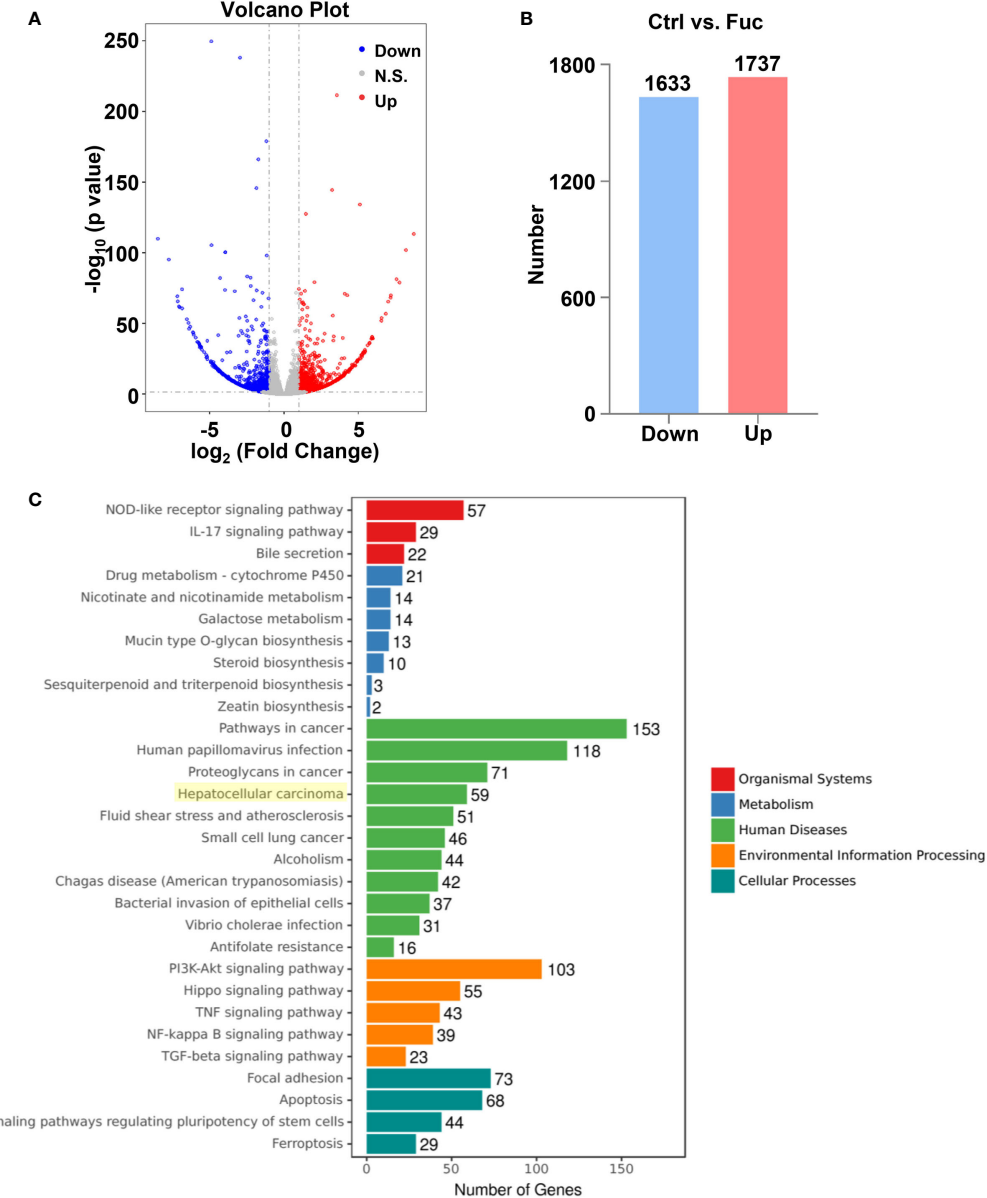
The alteration of mRNAs profiling regulated by fucoidan in MHCC-97H cells. **(A)** Volcano plots presents significantly different expression profiles of mRNAs in MHCC-97H cells treated with 0.5 mg/mL Fuc. **(B)** Numbers of significantly down and up-regulated mRNAs in MHCC-97H cells treated with 0.5 mg/mL Fuc. **(C)** Enriched KEGG pathways of differentially expressed mRNAs after fucoidan treatment. p value < 0.05.

To clarify the mechanism how fucoidan inhibits the development of HCC, we performed High-Throughput sequencing of lncRNAs in MHCC-97H cells with 0.5 mg/mL fucoidan treatment for 48 h. From the analysis of heatmap result, we could visually observe that large numbers of lncRNAs significantly altered. About 75% of all lncRNAs detected were previously annotated, while the remaining 25% were novel (**Figure 5A**). The detailed lncRNA names were listed in **Supplementary Tables S1** and **S2** (as shown in **Supplementary Material**). A total of 105 differentially expressed lncRNAs (DElncRNA) was identified with thresholds of log_2_ (Fold Change)>1 and adjusted P<0.05, of which 49 were down-regulated and 56 were up-regulated as presented by Volcano Plot result (**Figures 5B, C**). To better comprehend the underlying mechanisms of these DElncRNAs in the tumorigenesis of HCC, we performed mRNA sequencing. Volcano plots diagram showed that 1633 mRNAs were down-regulated and 1737 mRNAs were up-regulated (**Figures 6A, B**). We further conducted KEGG pathway analysis, which suggested that downstream mRNAs regulated by lncRNA closely related to HCC. In addition, the apoptosis-relevant genes were significantly changed after treated with 0.5 mg/mL fucoidan in MHCC-97H cells (**Figure 6C**).

### Fucoidan Obviously Increases the Expression Level of LINC00261 to Inhibit the Proliferation and Invasion of HCC Cells

**Figure 7:**
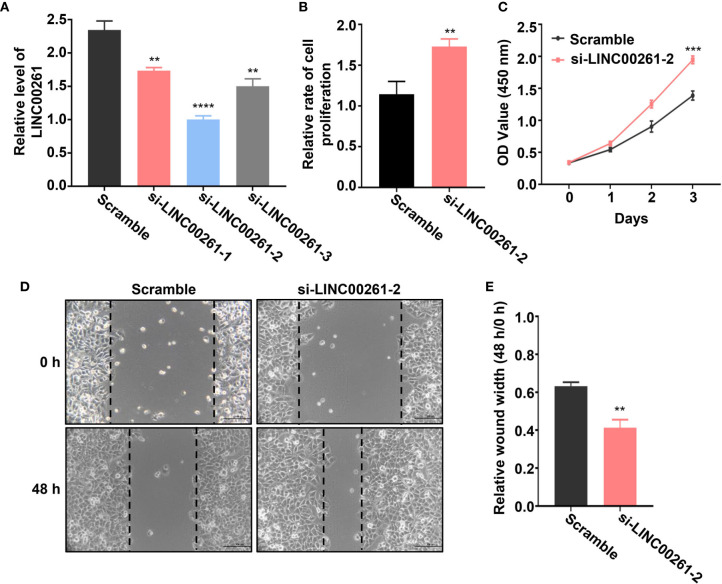
**(A)** Relative LINC00261 expression level in MHCC-97H cells detected by qPCR after transfected with scramble siRNA, si-LINC00261-1, si-LINC00261-2 and si-LINC00261-3. ** means P < 0.01, **** means P < 0.0001. **(B)** Relative cell proliferation of MHCC-97H cells transfected with scramble siRNA and siLINC00261-2. ** means P < 0.01. **(C)** Cell viability of MHCC-97H transfected with scramble siRNA and si-LINC00261-2. *** means P < 0.001. **(D, E)** Wound healing results of MHCC-97H transfected with scramble siRNA and si-LINC00261-2. ** means P < 0.01.

To further verify whether these DElncRNAs play an important role in HCC tumorigenesis, we selected a lncRNA named LINC00261 from all the differentially expressed lncRNAs. LINC00261 was confirmed by other studies to act as a tumor suppressor gene in prostate cancer, breast cancer, pancreatic cancer and many other types of cancers. For example, LINC00261 was found to inhibit lung cancer cells by interfering with the expression of downstream miR-1269a (55). Another research also confirmed that LINC00261 could inhibit the transcription of c-Myc in pancreatic cancer, thereby inhibiting the proliferation and metastasis of pancreatic cancer cells (56). According to our sequencing results, LINC00261 was significantly up-regulated in fucoidan treated group (log_2_ (fold change)=3.45). Since the anti-tumor effect of LINC00261 in HCC was rarely reported, we chose LINC00261 as the target of our further study. To explore the role of LINC00261 in HCC tumorigenesis, si-LINC00261 (si-LINC00261-1, si-LINC00261-2 and si-LINC00261-3) were respectively transfected in MHCC-97H cells. Scramble siRNA transfection was used as negative control. Then we used qPCR to detect the relative mRNA expression level of LINC00261 in MHCC-97H cells. The results showed that the expression level of LINC00261 decreased after transfection of three kinds of siRNAs and the knockdown effect of si-LINC00261-2 was the most significant. Then si-LINC00261-2 was selected for the follow-up experiments (**Figure 7A**). To demonstrate the effect of LINC00261 on cell proliferation and viability, we conducted the cell proliferation and viability assay after si-LINC00261-2 transfection. As shown in **Figure 7B**, after LINC00261 was knocked down, the relative proliferation rate of MHCC-97H cells was significantly increased than that of negative control group, and CCK-8 experiment also proved that HCC cell viability was improved after si-LINC00261-2 was transfected (**Figure 7C**). In addition, wound healing assay was carried out in MHCC-97H cells transfected with scrambled siRNA and si-LINC00261-2 to investigate the effect of LINC00261 on the motility of HCC cells. Results revealed that LINC00261 also inhibit the motility of HCC cells (**Figures 7D, E**). Based on the above results, we testified that fucoidan is able to increase the expression level of LINC00261, which plays an anti-tumor role by inhibiting the proliferation, viability and motility of HCC cells.

### LINC00261 Inhibits Proliferation of MHCC-97H Cells *via* Regulating miR-522-3p

**Figure 8:**
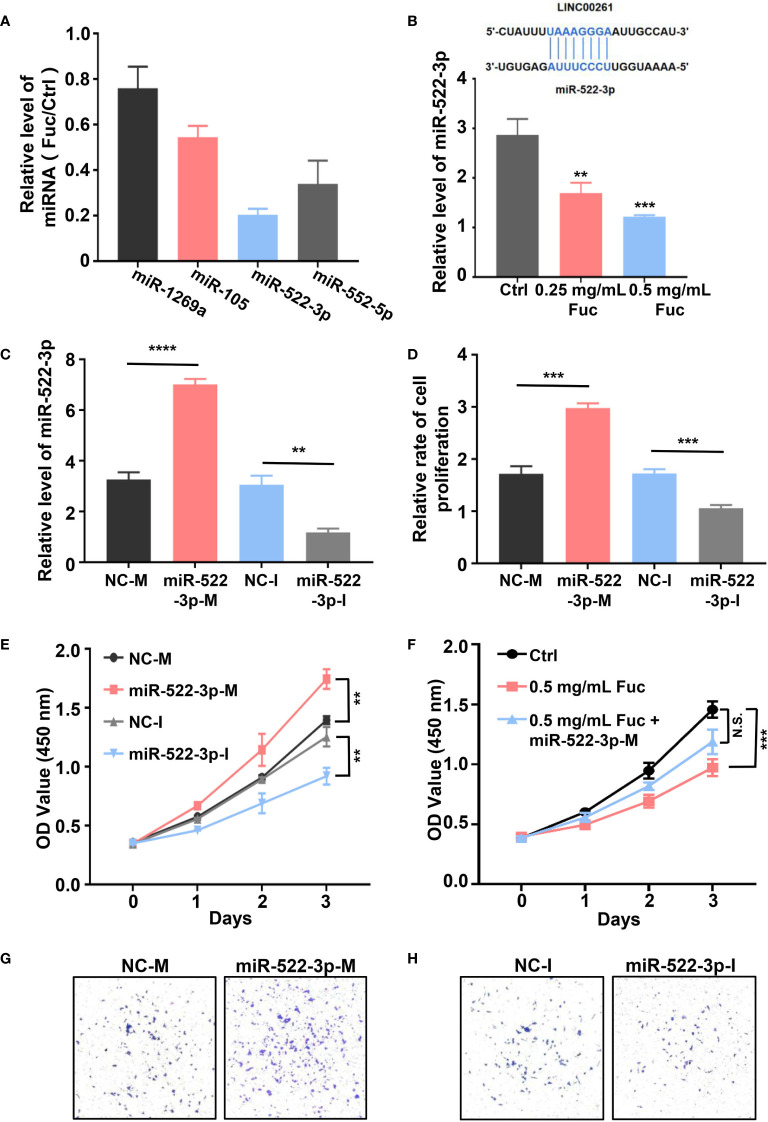
**(A)** The relative level of miRNAs equals to the level of miRNAs in Fuc group divided by the level of miRNAs in Ctrl group. **(B)** The schematic of LINC00261 binds to miR-522-3p by base pairing. The examination of miR-522-3p level in MHCC-97H cells treated with saline (Ctrl), 0.25 mg/mL Fuc and 0.5 mg/mL Fuc. ** means P < 0.01, *** means P < 0.001. **(C)** The qPCR results of MHCC-97H cells transfected with miR-522-3p mimic and inhibitor. NC-M is short for negative control of miRNA mimic. NC-I is short for negative control of miRNA inhibitor. ** means P < 0.01, **** means P < 0.0001. **(D)** Relative cell proliferation rate of MHCC-97H cells transfected with miR-522-3p mimic and inhibitor. *** means P < 0.001. **(E)** Cell viability of MHCC-97H cells transfected with miR-522-3p mimic and inhibitor. ** means P < 0.01. **(F)** The cell viability of MHCC-97H cells in Ctrl group, 0.5 mg/mL Fuc treated group, 0.5 mg/mL Fuc treated and miR-522-3p mimic (miR-522-3p-M) transfected group. *** means P < 0.001. **(G)** Overexpression of miR-522-3p accelerates cell invasion of MHCC-97H cells. **(H)** Inhibition of miR-522-3p restrained cell invasion of MHCC-97H cells.

To explore whether LINC00261 could target miRNAs in MHCC-97H cells, we examined the expression level of miR-1296a, miR-105, miR-522-3p and miR-552-5p (55, 57–59), which were reported to bind LINC00261. The qPCR results suggested that miR-522-3p was remarkably decreased by comparing fucoidan treated group with the Ctrl group (**Figure 8A**). Then we added equivalent saline, 0.25 mg/mL fucoidan and 0.5 mg/mL fucoidan into MHCC-97H cells and examined the expression level of miR-522-3p by qPCR. The results showed that fucoidan down-regulated miR-522-3p in dose-dependent manner (**Figure 8B**). MiR-522-3p mimic and inhibitor synthesized from GenePharma were separately transfected in MHCC-97H cells (**Figure 8C**). The results suggested that miR-522-3p mimic increased and miR-522-3p inhibitor decreased the proliferation rate of MHCC-97H cells (**Figure 8D**). Besides, we also examined the cell viability by CCK-8 assay and discovered that miR-522-3p mimic promoted, miR-522-3p inhibitor inhibited the cell viability of HCC cells (**Figure 8E**). To further address the mechanism of fucoidan inhibiting the cell viability of HCC cells by regulating miR-522-3p. MHCC-97H cells were transfected with miR-522-3p mimic and exposed to 0.5 mg/mL fucoidan at the same time. Compared with the only 0.5 mg/mL fucoidan treated group, miR-522-3p effectively rescued the inhibition of cell viability by fucoidan (**Figure 8F**). The transwell assay results further showed that overexpressing miR-522-3p promotes cell invasion of HCC and knocking down miR-522-3p inhibits cell invasion of HCC (**Figures 8G, H**). In conclusion, we demonstrated that fucoidan was able to increase the expression level of LINC00261, which inhibit cell proliferation and invasion of HCC *via* down-regulating miR-522-3p.

**Figure 9:**
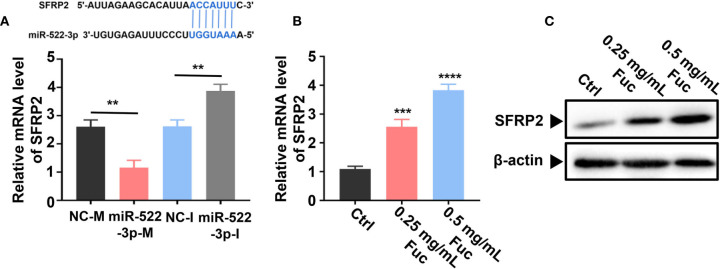
Fucoidan regulates SFRP2 *via* miR-522-3p. **(A)** miR-522-3p binds to SFRP2 by base pairing. miR-522-3p-M down-, miR-522-3p-I up-regulates the expression level of SFRP2. ** means P < 0.01. **(B)** Fucoidan obviously increases the level of SFRP2. *** means P < 0.001, **** means P < 0.0001. **(C)** Western blots analysis of SFRP2 in MHCC-97H cells treated with different dosages of fucoidan (0, 0.25, 0.5 mg/mL).

Previous studies (58) demonstrated that miR-522-3p binds to Wnt signaling related gene SFRP2 (secreted frizzled-related protein 2) by base pairing. The qPCR result suggested that miR-522-3p could inhibit the expression level of SFRP2 (**Figure 9A**). Intriguingly, fucoidan increased the expression level of SFRP2 in a dose-dependent manner, which indicated that fucoidan up-regulates LINC00261 sponging miR-522-3p to increase the expression level of SFRP2 (**Figure 9B**). Western blots analysis also verified that fucoidan obviously increased the protein level of SFRP2 (**Figure 9C**).

## Discussion

Safe and effective treatment has always been a key issue in the field of clinical treatment. Although the research and development of various chemical and biological agents and immunotherapy already made some previously incurable diseases cured in recent years, therapeutic drugs with less side effects and toxicity still need to be further explored. Natural compounds were widely used in food, cosmetics and other industries for many years. Because of their high safety, many clinical and preclinical studies begun to pay attention to them (60–64). As a natural polysaccharide extracted from brown algae, fucoidan was proved to have many biological effects (12–16). The anti-tumor abilities of fucoidan were confirmed in pancreatic cancer, bladder cancer, ovarian cancer and other types of cancers (65–68). Fucoidan could inhibit the occurrence and development of tumor by regulating tumor immunity (21, 22), inhibiting angiogenesis (23), interfering with cell cycle and apoptosis (19, 20), which is considered to have a broad prospect in the field of tumor therapy.

HCC has become the leading cause of cancer-related death worldwide (2). In view of the high incidence rate and mortality rate of HCC, diagnosis and treatment have always been a hot topic (3). Currently, the main treatment of HCC includes surgery, traditional chemotherapy drugs and novel targeted drugs (4). Although these therapeutic methods improved the prognosis and survival rate of HCC patients to a certain extent, many patients still die of HCC due to the limited therapeutic effects. Therefore, further exploration of novel treatments is necessary. Since the application of fucoidan in the treatment of HCC is rarely reported, our study focused on the anti-tumor effect of fucoidan in HCC. According to the experimental results, fucoidan affects HCC tumorigenesis through inhibiting proliferation, motility and invasion of MHCC-97H cells and it also plays an important role in arresting cell cycle and promoting cell apoptosis in a dose-dependent manner.

lncRNAs were proved to be involved in a variety of biological activities, which is of great significance in regulating cell growth, differentiation and characteristics. They were also testified to play roles in the regulation of various types of tumors (50–53). In this paper, we took advantage of High-Throughout sequencing to reveal the changes of lncRNA expression profiles in HCC after fucoidan treatment. Among the detected lncRNAs, 75% of which were previously reported and the other lncRNAs were novel. Further KEGG analysis show that these up-regulated and down-regulated lncRNAs were involved in cellular biological functions and intracellular signal pathways in HCC development. Hence, we suggest that fucoidan play an anti-tumor effect by regulating the expression level of lncRNAs. In our study, we select a lncRNA named LINC00261, which was proved in other studies to play an anti-tumor role by regulating the expression of downstream microRNA (59, 69, 70). We proved that LINC00261 could inhibit the tumor characteristics of MHCC-97H cells by regulating miR-522-3p in HCC. Shi et al. reported that miR-522-3p interacted with SFRP2 to suppress Wnt signaling pathway in non–small cell lung cancer (58). In our study, we found miR-522-3p indeed down-regulated the expression level of SFRP2. We examined the mRNA and protein level of SFRP2 in MHCC-97H cells treated with saline, 0.25 and 0.5 mg/mL fucoidan. Interestingly, fucoidan markedly promoted the expression level of SFRP2, which indicated the LINC00261- miR-522-3p- SFRP2 interacting effects in HCC cells. Although some other novel lncRNAs were also detected, lncRNAs could also affect tumorigenesis by regulating the expression of tumor suppressor genes, activating intracellular signaling pathways, regulating cell cycle and other ways according to other studies (28, 36, 44, 46, 48), these lncRNAs involved in the fucoidan regulatory pathway and their underlying regulatory mechanisms require further study, which may contribute greatly to the future HCC therapy.

## Data Availability Statement

The datasets presented in this study can be found in online repositories. The names of the repository/repositories and accession number(s) can be found below: https://www.ncbi.nlm.nih.gov/, PRJNA690771.

## Ethics Statement

The animal study was reviewed and approved by Animal Care and Use Committee, Zhongshan Hospital of Fudan University.

## Author Contributions

DM, JW, SC, and HW conducted the experiments, analyzed data, and wrote the manuscript. LN, and S-HL conducted the experiments. C-LL contributed reagents. QY and GS provided the concept, designed the study, interpreted the results and wrote the manuscript. All authors contributed to the article and approved the submitted version.

## Funding

This work was supported by the grant from the National Natural Science Foundation of China (No. 81700550 to GS and No. 81500460 to QY), by the Natural Science Foundation of Shanghai (No. 20ZR1411200 to GS), by the grant from the Zhongshan Hospital of Fudan University (No. H2020-064).

## Conflict of Interest

C-LL is employee of Hi-Q Marine Biotech International Ltd.

The remaining authors declare that the research was conducted in the absence of any commercial or financial relationships that could be construed as a potential conflict of interest.

## References

[1] Bray F Ferlay J Soerjomataram I Siegel RL Torre LA Jemal A. Global cancer statistics 2018: GLOBOCAN estimates of incidence and mortality worldwide for 36 cancers in 185 countries. CA Cancer J Clin (2018) 68:394–424. 10.3322/caac.21492 30207593

[2] Sayiner M Golabi P Younossi ZM. Disease Burden of Hepatocellular Carcinoma: A Global Perspective. Dig Dis Sci (2019) 64:910–7. 10.1007/s10620-019-05537-2 30835028

[3] Petrick JL Kelly SP Altekruse SF McGlynn KA Rosenberg PS. Future of Hepatocellular Carcinoma Incidence in the United States Forecast Through 2030. J Clin Oncol (2016) 34:1787–94. 10.1200/jco.2015.64.7412 PMC4966339 27044939

[4] Grandhi MS Kim AK Ronnekleiv-Kelly SM Kamel IR Ghasebeh MA Pawlik TM. Hepatocellular carcinoma: From diagnosis to treatment. Surg Oncol (2016) 25:74–85. 10.1016/j.suronc.2016.03.002 27312032

[5] Li H Wu F Duan M Zhang G. Drug-eluting bead transarterial chemoembolization (TACE) vs conventional TACE in treating hepatocellular carcinoma patients with multiple conventional TACE treatments history: A comparison of efficacy and safety. Med (Baltimore) (2019) 98:e15314. 10.1097/md.0000000000015314 PMC6571239 31124925

[6] Tsurusaki M Murakami T. Surgical and Locoregional Therapy of HCC: TACE. Liver Cancer (2015) 4:165–75. 10.1159/000367739 PMC4608659 26675172

[7] Nault JC Galle PR Marquardt JU. The role of molecular enrichment on future therapies in hepatocellular carcinoma. J Hepatol (2018) 69:237–47. 10.1016/j.jhep.2018.02.016 29505843

[8] Heinrich B Czauderna C Marquardt JU. Immunotherapy of Hepatocellular Carcinoma. Oncol Res Treat (2018) 41:292–7. 10.1159/000488916 29705790

[9] Fitton HJ Stringer DS Park AY Karpiniec SN. Therapies from Fucoidan: New Developments. Mar Drugs (2019) 17:571. 10.3390/md17100571 PMC6836154 31601041

[10] Wang J Geng L Yue Y Zhang Q. Use of fucoidan to treat renal diseases: A review of 15 years of clinic studies. Prog Mol Biol Transl Sci (2019) 163:95–111. 10.1016/bs.pmbts.2019.03.011 31030763

[11] Luthuli S Wu S Cheng Y Zheng X Wu M Tong H. Therapeutic Effects of Fucoidan: A Review on Recent Studies. Mar Drugs (2019) 17:487. 10.3390/md17090487 PMC6780838 31438588

[12] Wang Y Xing M Cao Q Ji A Liang H Song S. Biological Activities of Fucoidan and the Factors Mediating Its Therapeutic Effects: A Review of Recent Studies. Mar Drugs (2019) 17:183. 10.3390/md17030183 PMC6471298 30897733

[13] Wu L Sun J Su X Yu Q Yu Q Zhang P. A review about the development of fucoidan in antitumor activity: Progress and challenges. Carbohydr Polym (2016) 154:96–111. 10.1016/j.carbpol.2016.08.005 27577901

[14] Palanisamy S Vinosha M Manikandakrishnan M Anjali R Rajasekar P Marudhupandi T. Investigation of antioxidant and anticancer potential of fucoidan from Sargassum polycystum. Int J Biol Macromol (2018) 116:151–61. 10.1016/j.ijbiomac.2018.04.163 29729339

[15] Mansour MB Balti R Yacoubi L Ollivier V Chaubet F Maaroufi RM. Primary structure and anticoagulant activity of fucoidan from the sea cucumber Holothuria polii. Int J Biol Macromol (2019) 121:1145–53. 10.1016/j.ijbiomac.2018.10.129 30340002

[16] Elizondo-Gonzalez R Cruz-Suarez LE Ricque-Marie D Mendoza-Gamboa E Rodriguez-Padilla C Trejo-Avila LM. In vitro characterization of the antiviral activity of fucoidan from Cladosiphon okamuranus against Newcastle Disease Virus. Virol J (2012) 9:307. 10.1186/1743-422x-9-307 23234372 PMC3546940

[17] Wu SY Yang WY Cheng CC Lin KH Sampurna BP Chan SM. Low molecular weight fucoidan inhibits hepatocarcinogenesis and nonalcoholic fatty liver disease in zebrafish via ASGR/STAT3/HNF4A signaling. Clin Transl Med (2020) 10:e252. 10.1002/ctm2.252 33377648 PMC7752165

[18] Atashrazm F Lowenthal RM Woods GM Holloway AF Dickinson JL. Fucoidan and cancer: a multifunctional molecule with anti-tumor potential. Mar Drugs (2015) 13:2327–46. 10.3390/md13042327 PMC4413214 25874926

[19] Zhang Z Teruya K Eto H Shirahata S. Fucoidan extract induces apoptosis in MCF-7 cells via a mechanism involving the ROS-dependent JNK activation and mitochondria-mediated pathways. PloS One (2011) 6:e27441. 10.1371/journal.pone.0027441 22096572 PMC3214060

[20] Kim IH Kwon MJ Nam TJ. Differences in cell death and cell cycle following fucoidan treatment in high-density HT-29 colon cancer cells. Mol Med Rep (2017) 15:4116–22. 10.3892/mmr.2017.6520 PMC5436236 28487956

[21] Zhang W Oda T Yu Q Jin JO. Fucoidan from Macrocystis pyrifera has powerful immune-modulatory effects compared to three other fucoidans. Mar Drugs (2015) 13:1084–104. 10.3390/md13031084 PMC4377974 25706632

[22] Cheng CC Yang WY Hsiao MC Lin KH Lee HW Yuh CH. Transcriptomically Revealed Oligo-Fucoidan Enhances the Immune System and Protects Hepatocytes via the ASGPR/STAT3/HNF4A Axis. Biomolecules (2020) 10:898. 10.3390/biom10060898 PMC7355575 32545625

[23] Rui X Pan HF Shao SL Xu XM. Anti-tumor and anti-angiogenic effects of Fucoidan on prostate cancer: possible JAK-STAT3 pathway. BMC Complement Altern Med (2017) 17:378. 10.1186/s12906-017-1885-y 28764703 PMC5540291

[24] Carninci P Kasukawa T Katayama S Gough J Frith MC Maeda N. The transcriptional landscape of the mammalian genome. Science (2005) 309:1559–63. 10.1126/science.1112014 16141072

[25] Jathar S Kumar V Srivastava J Tripathi V. Technological Developments in lncRNA Biology. Adv Exp Med Biol (2017) 1008:283–323. 10.1007/978-981-10-5203-3_10 28815544

[26] Guo X Gao L Wang Y Chiu DK Wang T Deng Y. Advances in long noncoding RNAs: identification, structure prediction and function annotation. Brief Funct Genomics (2016) 15:38–46. 10.1093/bfgp/elv022 26072035 PMC5863772

[27] Sun TT He J Liang Q Ren LL Yan TT Yu TC. LncRNA GClnc1 Promotes Gastric Carcinogenesis and May Act as a Modular Scaffold of WDR5 and KAT2A Complexes to Specify the Histone Modification Pattern. Cancer Discovery (2016) 6:784–801. 10.1158/2159-8290.Cd-15-0921 27147598

[28] Mondal T Subhash S Vaid R Enroth S Uday S Reinius B. MEG3 long noncoding RNA regulates the TGF-β pathway genes through formation of RNA-DNA triplex structures. Nat Commun (2015) 6:7743. 10.1038/ncomms8743 26205790 PMC4525211

[29] Kino T Hurt DE Ichijo T Nader N Chrousos GP. Noncoding RNA gas5 is a growth arrest- and starvation-associated repressor of the glucocorticoid receptor. Sci Signal (2010) 3:ra8. 10.1126/scisignal.2000568 20124551 PMC2819218

[30] Thomson DW Dinger ME. Endogenous microRNA sponges: evidence and controversy. Nat Rev Genet (2016) 17:272–83. 10.1038/nrg.2016.20 27040487

[31] Chan JJ Tay Y. Noncoding RNA:RNA Regulatory Networks in Cancer. Int J Mol Sci (2018) 19:1310. 10.3390/ijms19051310 PMC5983611 29702599

[32] Bartonicek N Maag JL Dinger ME. Long noncoding RNAs in cancer: mechanisms of action and technological advancements. Mol Cancer (2016) 15:43. 10.1186/s12943-016-0530-6 27233618 PMC4884374

[33] Vitiello M Tuccoli A Poliseno L. Long non-coding RNAs in cancer: implications for personalized therapy. Cell Oncol (Dordr) (2015) 38:17–28. 10.1007/s13402-014-0180-x 25113790 PMC13004270

[34] Bhan A Soleimani M Mandal SS. Long Noncoding RNA and Cancer: A New Paradigm. Cancer Res (2017) 77:3965–81. 10.1158/0008-5472.Can-16-2634 PMC8330958 28701486

[35] Peng WX Koirala P Mo YY. LncRNA-mediated regulation of cell signaling in cancer. Oncogene (2017) 36:5661–7. 10.1038/onc.2017.184 PMC6450570 28604750

[36] Barsyte-Lovejoy D Lau SK Boutros PC Khosravi F Jurisica I Andrulis IL. The c-Myc oncogene directly induces the H19 noncoding RNA by allele-specific binding to potentiate tumorigenesis. Cancer Res (2006) 66:5330–7. 10.1158/0008-5472.Can-06-0037 16707459

[37] Liu C Chen L You Z Wu Y Wang C Zhang G. Association between lncRNA H19 polymorphisms and cancer susceptibility based on a meta-analysis from 25 studies. Gene (2020) 729:144317. 10.1016/j.gene.2019.144317 31884107

[38] Ye Y Shen A Liu A. Long non-coding RNA H19 and cancer: A competing endogenous RNA. Bull Cancer (2019) 106:1152–9. 10.1016/j.bulcan.2019.08.011 31753509

[39] Bhan A Mandal SS. LncRNA HOTAIR: A master regulator of chromatin dynamics and cancer. Biochim Biophys Acta (2015) 1856:151–64. 10.1016/j.bbcan.2015.07.001 PMC4544839 26208723

[40] Loewen G Jayawickramarajah J Zhuo Y Shan B. Functions of lncRNA HOTAIR in lung cancer. J Hematol Oncol (2014) 7:90. 10.1186/s13045-014-0090-4 25491133 PMC4266198

[41] Tang Q Hann SS. HOTAIR: An Oncogenic Long Non-Coding RNA in Human Cancer. Cell Physiol Biochem (2018) 47:893–913. 10.1159/000490131 29843138

[42] Gutschner T Hämmerle M Eissmann M Hsu J Kim Y Hung G. The noncoding RNA MALAT1 is a critical regulator of the metastasis phenotype of lung cancer cells. Cancer Res (2013) 73:1180–9. 10.1158/0008-5472.Can-12-2850 PMC3589741 23243023

[43] Li ZX Zhu QN Zhang HB Hu Y Wang G Zhu YS. MALAT1: a potential biomarker in cancer. Cancer Manag Res (2018) 10:6757–68. 10.2147/cmar.S169406 PMC6289210 30584369

[44] Wu Q Meng WY Jie Y Zhao H. LncRNA MALAT1 induces colon cancer development by regulating miR-129-5p/HMGB1 axis. J Cell Physiol (2018) 233:6750–7. 10.1002/jcp.26383 PMC13482480 29226325

[45] Yao W Bai Y Li Y Guo L Zeng P Wang Y. Upregulation of MALAT-1 and its association with survival rate and the effect on cell cycle and migration in patients with esophageal squamous cell carcinoma. Tumour Biol (2016) 37:4305–12. 10.1007/s13277-015-4223-3 26493997

[46] Chen J Wu D Zhang Y Yang Y Duan Y An Y. LncRNA DCST1-AS1 functions as a competing endogenous RNA to regulate FAIM2 expression by sponging miR-1254 in hepatocellular carcinoma. Clin Sci (Lond) (2019) 133:367–79. 10.1042/cs20180814 30617187

[47] Castro-Oropeza R Melendez-Zajgla J Maldonado V Vazquez-Santillan K. The emerging role of lncRNAs in the regulation of cancer stem cells. Cell Oncol (Dordr) (2018) 41:585–603. 10.1007/s13402-018-0406-4 30218296 PMC12995221

[48] Hu M Zhang Q Tian XH Wang JL Niu YX Li G. lncRNA CCAT1 is a biomarker for the proliferation and drug resistance of esophageal cancer via the miR-143/PLK1/BUBR1 axis. Mol Carcinog (2019) 58:2207–17. 10.1002/mc.23109 31544294

[49] Yang Y Jiang C Yang Y Guo L Huang J Liu X. Silencing of LncRNA-HOTAIR decreases drug resistance of Non-Small Cell Lung Cancer cells by inactivating autophagy via suppressing the phosphorylation of ULK1. Biochem Biophys Res Commun (2018) 497:1003–10. 10.1016/j.bbrc.2018.02.141 29470986

[50] Yu X Cao Y Tang L Yang Y Chen F Xia J. Baicalein inhibits breast cancer growth via activating a novel isoform of the long noncoding RNA PAX8-AS1-N. J Cell Biochem (2018) 119:6842–56. 10.1002/jcb.26881 29693272

[51] Wang M Jiang S Yu F Zhou L Wang K. Noncoding RNAs as Molecular Targets of Resveratrol Underlying Its Anticancer Effects. J Agric Food Chem (2019) 67:4709–19. 10.1021/acs.jafc.9b01667 30990036

[52] Liu G Xiang T Wu QF Wang WX. Curcumin suppresses the proliferation of gastric cancer cells by downregulating H19. Oncol Lett (2016) 12:5156–62. 10.3892/ol.2016.5354 PMC5228417 28105222

[53] Zhou B Yu Y Yu L Que B Qiu R. Sipi soup inhibits cancer−associated fibroblast activation and the inflammatory process by downregulating long non−coding RNA HIPK1−AS. Mol Med Rep (2018) 18:1361–8. 10.3892/mmr.2018.9144 PMC6072218 29901171

[54] Hsu HY Hwang PA. Clinical applications of fucoidan in translational medicine for adjuvant cancer therapy. Clin Transl Med (2019) 8:15. 10.1186/s40169-019-0234-9 31041568 PMC6491526

[55] Guo C Shi H Shang Y Zhang Y Cui J Yu H. LncRNA LINC00261 overexpression suppresses the growth and metastasis of lung cancer via regulating miR-1269a/FOXO1 axis. Cancer Cell Int (2020) 20:275. 10.1186/s12935-020-01332-6 32607060 PMC7318380

[56] Liu S Zheng Y Zhang Y Zhang J Xie F Guo S. Methylation-mediated LINC00261 suppresses pancreatic cancer progression by epigenetically inhibiting c-Myc transcription. Theranostics (2020) 10:10634–51. 10.7150/thno.44278 PMC7482811 32929371

[57] Chen T Lei S Zeng Z Zhang J Xue Y Sun Y. Linc00261 inhibits metastasis and the WNT signaling pathway of pancreatic cancer by regulating a miR−552−5p/FOXO3 axis. Oncol Rep (2020) 43:930–42. 10.3892/or.2020.7480 PMC7041108 32020223

[58] Shi J Ma H Wang H Zhu W Jiang S Dou R. Overexpression of LINC00261 inhibits non-small cell lung cancer cells progression by interacting with miR-522-3p and suppressing Wnt signaling. J Cell Biochem (2019) 120:18378–87. 10.1002/jcb.29149 31190356

[59] Wang Z Zhang J Yang B Li R Jin L Wang Z. Long Intergenic Noncoding RNA 00261 Acts as a Tumor Suppressor in Non-Small Cell Lung Cancer via Regulating miR-105/FHL1 Axis. J Cancer (2019) 10:6414–21. 10.7150/jca.32251 PMC6856729 31772674

[60] Cornejo A Caballero J Simirgiotis M Torres V Sánchez L Díaz N. Dammarane triterpenes targeting α-synuclein: biological activity and evaluation of binding sites by molecular docking. J Enzyme Inhib Med Chem (2021) 36:154–62. 10.1080/14756366.2020.1851216 PMC7738290 33307873

[61] Huang Y Yuan K Tang M Yue J Bao L Wu S. Melatonin inhibiting the survival of human gastric cancer cells under ER stress involving autophagy and Ras-Raf-MAPK signalling. J Cell Mol Med (2020) 25:1480–92. 10.1111/jcmm.16237 PMC7875909 33369155

[62] Mohammadian Haftcheshmeh S Khosrojerdi A Aliabadi A Lotfi S Mohammadi A Momtazi-Borojeni AA. Immunomodulatory Effects of Curcumin in Rheumatoid Arthritis: Evidence from Molecular Mechanisms to Clinical Outcomes. Rev Physiol Biochem Pharmacol (2021). 10.1007/112_2020_54 33404796

[63] Wei C Xiao Q Kuang X Zhang T Yang Z Wang L. Fucoidan inhibits proliferation of the SKM-1 acute myeloid leukaemia cell line via the activation of apoptotic pathways and production of reactive oxygen species. Mol Med Rep (2015) 12:6649–55. 10.3892/mmr.2015.4252 PMC4626197 26324225

[64] Xiang Y Guo Z Zhu P Chen J Huang Y. Traditional Chinese medicine as a cancer treatment: Modern perspectives of ancient but advanced science. Cancer Med (2019) 8:1958–75. 10.1002/cam4.2108 PMC6536969 30945475

[65] Etman SM Mehanna RA Bary AA Elnaggar YSR Abdallah OY. Undaria pinnatifida fucoidan nanoparticles loaded with quinacrine attenuate growth and metastasis of pancreatic cancer. Int J Biol Macromol (2020) 170:284–97. 10.1016/j.ijbiomac.2020.12.109 33340624

[66] Jafari M Sriram V Xu Z Harris GM Lee JY. Fucoidan-Doxorubicin Nanoparticles Targeting P-Selectin for Effective Breast Cancer Therapy. Carbohydr Polym (2020) 249:116837. 10.1016/j.carbpol.2020.116837 32933681

[67] Liu S Yang J Peng X Li J Zhu C. The Natural Product Fucoidan Inhibits Proliferation and Induces Apoptosis of Human Ovarian Cancer Cells: Focus on the PI3K/Akt Signaling Pathway. Cancer Manag Res (2020) 12:6195–207. 10.2147/cmar.S254784 PMC7434378 32884336

[68] Miyata Y Matsuo T Ohba K Mitsunari K Mukae Y Otsubo A. Present Status, Limitations and Future Directions of Treatment Strategies Using Fucoidan-Based Therapies in Bladder Cancer. Cancers (Basel) (2020) 12:3776. 10.3390/cancers12123776 PMC7765304 33333858

[69] Gao J Qin W Kang P Xu Y Leng K Li Z. Up-regulated LINC00261 predicts a poor prognosis and promotes a metastasis by EMT process in cholangiocarcinoma. Pathol Res Pract (2020) 216:152733. 10.1016/j.prp.2019.152733 31812439

[70] Zhou Z Ma J. Expression and Clinical Significance of Long-chain Noncoding RNA LINC00261 in Colon Cancer. Clin Lab (2019) 65(12). 10.7754/Clin.Lab.2019.190402 31850713

