# Supplementary material for: Fucoidan Inhibits the Progression of Hepatocellular Carcinoma via Causing lncRNA LINC00261 Overexpression
Source: Front Oncol. 2021 Apr 13;11:653902. doi: 10.3389/fonc.2021.653902 (PMC8078595; doi:10.3389/fonc.2021.653902)
Supplement: Supplementary file 1 [file DataSheet_1.pdf]

frontiers

Supplementary Material

Fucoidan inhibits the progression of hepatocellular carcinoma via causing lncRNA LINC00261 overexpression

Danhui Ma[1, 2†], Jiayi Wei[1,2†], Sinuo Chen[1,2†], Heming Wang[1,2], Liuxin Ning[1,2], Shi-Hua Luo[3], Chieh-Lun Liu[4], Guangqi Song[1, 2*], Qunyan Yao[1,2*]

TABLE S1 | Up-regulated lncRNAs in MHCC-97H cells treated with 0.5 mg/mL Fucoidan.

Gene_ID	GeneSymbol	$Log_2FoldChange$	P value	Regulation
TCONS_00080504	POC1B-AS1	6.08268875	2.09634E-18	Ups
TCONS_00315956	LINC01184	5.785320922	2.79969E-16	Ups
TCONS_00359131	MET	5.029453204	3.76461E-11	Ups
TCONS_00366255	AC111149.2	4.585785196	2.8216E-14	Ups
TCONS_00359138	MET	4.137414696	2.29888E-07	Ups
TCONS_00070294	COLCA1	3.953667047	9.86224E-07	Ups
TCONS_00040980	OLMALINC	3.551683716	1.81611E-05	Ups
TCONS_00000107	LINC01128	3.534884312	1.85795E-05	Ups
TCONS_00238571	LINC00261	3.45168775	1.33313E-87	Ups
TCONS_00244495	MCM3AP-AS1	3.408603536	4.27241E-05	Ups
TCONS_00027726	-	3.24380467	5.61679E-05	Ups
TCONS_00383101	-	3.151628338	1.96234E-14	Ups
TCONS_00007611	ABCA4	3.14611394	5.46248E-13	Ups
TCONS_00383102	-	3.082637738	0.000254697	Ups
TCONS_00204213	-	3.080425475	0.000264414	Ups
TCONS_00242124	CU633906.2	2.993010476	0.000402803	Ups
TCONS_00383432	-	2.987364378	7.58851E-20	Ups
TCONS_00227536	HAGLR	2.87896642	0.00069929	Ups
TCONS_00378263	CASC19	2.87896642	0.00069929	Ups
TCONS_00338228	AL356124.1	2.876839001	4.04017E-06	Ups
TCONS_00169247	-	2.856620593	1.16108E-05	Ups
TCONS_00335815	LINC02535	2.807503036	0.000978925	Ups
TCONS_00007668	AC105942.1	2.731107025	0.001347182	Ups
TCONS_00070297	COLCA1	2.725314127	6.26569E-47	Ups
TCONS_00287765	LINC01091	2.718246128	0.000933172	Ups
TCONS_00254542	-	2.696249763	0.001576625	Ups
TCONS_00207516	-	2.688971968	0.001613305	Ups
TCONS_00201455	AC013442.1	2.684993813	0.001263793	Ups

**TCONS_00225337**	**AC016682.1**	**2.626544656**	**3.20449E-06**	**Ups**
**TCONS_00178171**	**LINC02864**	**2.605411584**	**0.002288351**	**Ups**
**TCONS_00201454**	**AC013442.1**	**2.409533797**	**0.001304137**	**Ups**
**TCONS_00189194**	**AC009005.1**	**2.383485203**	**0.001953996**	**Ups**
**TCONS_00222451**	**LINC00342**	**2.284167942**	**2.65883E-34**	**Ups**
**TCONS_00222719**	**AC092683.1**	**2.260130725**	**1.45402E-05**	**Ups**
**TCONS_00204231**	**AC007099.1**	**2.250770031**	**0.001563094**	**Ups**
**TCONS_00238570**	**LINC00261**	**2.166965425**	**5.27727E-05**	**Ups**
**TCONS_00055310**	**MALAT1**	**2.131932607**	**0.001532855**	**Ups**
**TCONS_00358301**	**AC007384.1**	**2.057871667**	**0.000546229**	**Ups**
**TCONS_00298679**	**AC105285.1**	**2.028985058**	**1.77327E-05**	**Ups**
**TCONS_00348068**	**LINC01393**	**1.977855001**	**8.96967E-06**	**Ups**
**TCONS_00305835**	**-**	**1.918736976**	**9.86217E-12**	**Ups**
**TCONS_00377761**	**SNTB1**	**1.806375137**	**2.27205E-11**	**Ups**
**TCONS_00268691**	**AC018816.1**	**1.728682473**	**3.47535E-10**	**Ups**
**TCONS_00382684**	**-**	**1.647912739**	**7.611E-07**	**Ups**
**TCONS_00221308**	**-**	**1.578818659**	**3.46764E-17**	**Ups**
**TCONS_00080506**	**POC1B-AS1**	**1.576718537**	**0.000406392**	**Ups**
**TCONS_00086332**	**-**	**1.486944486**	**0.001238324**	**Ups**
**TCONS_00205511**	**AC159540.2**	**1.451232096**	**8.08603E-05**	**Ups**
**TCONS_00178195**	**LINC02864**	**1.41157078**	**6.63349E-05**	**Ups**
**TCONS_00222467**	**LINC00342**	**1.404536139**	**9.41894E-27**	**Ups**
**TCONS_00283777**	**AC079921.1**	**1.339166048**	**1.42391E-06**	**Ups**
**TCONS_00069196**	**AP000648.9**	**1.303304357**	**0.000163067**	**Ups**
**TCONS_00344368**	**-**	**1.288934269**	**0.000271327**	**Ups**
**TCONS_00390752**	**-**	**1.277597077**	**1.30024E-21**	**Ups**
**TCONS_00222456**	**LINC00342**	**1.261007248**	**4.37279E-06**	**Ups**
**TCONS_00222212**	**-**	**1.259910894**	**0.0009043**	**Ups**
**TCONS_00359135**	**MET**	**1.258628759**	**2.97414E-10**	**Ups**
**TCONS_00080596**	**-**	**1.255730094**	**1.36302E-10**	**Ups**
**TCONS_00222472**	**LINC00342**	**1.190353148**	**6.93228E-10**	**Ups**
**TCONS_00056343**	**MYEOV**	**1.159354236**	**0.000333152**	**Ups**
**TCONS_00405834**	**-**	**1.147894348**	**7.48769E-05**	**Ups**
**TCONS_00222160**	**-**	**1.135742965**	**0.00130752**	**Ups**
**TCONS_00411392**	**FIRRE**	**1.118223255**	**1.65036E-07**	**Ups**
**TCONS_00341856**	**AC073332.1**	**1.106100335**	**1.57629E-05**	**Ups**
**TCONS_00222113**	**-**	**1.087781559**	**0.000480164**	**Ups**
**TCONS_00222352**	**-**	**1.077208471**	**0.0020076**	**Ups**

**TABLE S2 | ** Down-regulated lncRNAs in MHCC-97H cells treated with 0.5 mg/mL Fucoidan.

Gene_ID	GeneSymbol	Log_2FoldChange	P value	Regulation
TCONS_00397442	-	-8.449722129	1.19959E-43	Down
TCONS_00404686	LINC00894	-4.670547638	1.68733E-09	Down
TCONS_00193598	LINC00662	-4.623051671	3.63482E-09	Down
TCONS_00397179	-	-4.033057545	6.2796E-202	Down
TCONS_00340912	AC091729.3	-3.659562166	9.3146E-06	Down
TCONS_00360067	AC009275.1	-3.46250075	3.67441E-05	Down
TCONS_00314283	ANT2	-3.392730419	4.93951E-05	Down
TCONS_00141416	-	-3.301894869	8.24361E-05	Down
TCONS_00380062	-	-3.22603176	0.000127742	Down
TCONS_00093856	LINC01234	-3.146757107	0.000192515	Down
TCONS_00033577	LYPLAL1-DT	-3.128686556	0.000213898	Down
TCONS_00142911	ERVK13-1	-3.108463048	0.000235512	Down
TCONS_00397440	-	-2.829267283	2.26163E-44	Down
TCONS_00033566	LYPLAL1-DT	-2.780225173	2.25988E-05	Down
TCONS_00159677	-	-2.684752764	0.000136634	Down
TCONS_00331515	-	-2.684491061	0.001670724	Down
TCONS_00221840	AC133644.1	-2.649658397	0.001943162	Down
TCONS_00132407	AC036108.2	-2.637753919	8.23318E-10	Down
TCONS_00397175	-	-2.626897121	1.96314E-83	Down
TCONS_00354676	LINC01446	-2.624313631	0.001841216	Down
TCONS_00080583	AC025254.1	-2.613572302	0.002257404	Down
TCONS_00309825	-	-2.471565073	0.000598051	Down
TCONS_00084319	AC007406.3	-2.386472677	3.39144E-05	Down
TCONS_00397439	-	-2.267000067	8.49491E-65	Down
TCONS_00133468	AC005606.1	-2.253350504	8.15915E-12	Down
TCONS_00132406	AC036108.2	-2.199653542	1.46541E-10	Down
TCONS_00221327	-	-2.180666758	0.000901745	Down
TCONS_00132444	AC015660.1	-2.16152284	0.00010174	Down
TCONS_00397441	-	-2.132815487	3.09303E-43	Down
TCONS_00282054	NOP14-AS1	-2.052233401	1.71252E-09	Down
TCONS_00369159	PVT1	-1.891800517	0.002037211	Down
TCONS_00285069	GC	-1.853995142	0.002197654	Down
TCONS_00368689	AC090192.2	-1.853396321	1.90769E-13	Down
TCONS_00215972	ID2-AS1	-1.8250015	0.001074723	Down
TCONS_00083217	AC027290.1	-1.755978188	5.39782E-06	Down
TCONS_00233935	-	-1.693488426	8.46792E-09	Down
TCONS_00397445	-	-1.673982973	1.00301E-20	Down
TCONS_00028995	BX470102.1	-1.667550219	2.53275E-06	Down
TCONS_00397186	-	-1.634063126	1.86166E-28	Down
TCONS_00330102	-	-1.566342117	2.94469E-12	Down
TCONS_00093140	CRY1	-1.504991392	4.59733E-05	Down

**TCONS_00397446**	**-**	**-1.454823282**	**3.32491E-22**	**Down**
**TCONS_00061371**	**H19**	**-1.427247666**	**7.64177E-27**	**Down**
**TCONS_00410722**	**DANT2**	**-1.229923382**	**5.78237E-09**	**Down**
**TCONS_00233938**	**-**	**-1.173605783**	**4.87711E-07**	**Down**
**TCONS_00265299**	**RNU2-31P**	**-1.164605407**	**0.000194798**	**Down**
**TCONS_00031281**	**GAS5**	**-1.162151149**	**0.000196551**	**Down**
**TCONS_00299608**	**SLC9A3-AS1**	**-1.147920532**	**0.000865497**	**Down**
**TCONS_00232669**	**-**	**-1.081455746**	**0.000113068**	**Down**
**TCONS_00159478**	**AC132872.3**	**-1.034507237**	**9.84862E-12**	**Down**
**TCONS_00359111**	**AC006159.1**	**-1.022247312**	**3.07912E-05**	**Down**

TABLE S3 | The sequences of primers used in this paper.

**Primer name**	**Sequence(5'-3')**
**GAPDH-F**	**AGCCACATCGCTCAGACAC**
**GAPDH-R**	**GCCCAATACGACCAAATCC**
**LINC00261-F**	**ACATTTGGTAGCCCGTGGAG**
**LINC00261-R**	**TCTTCCCCGGAGAACTAGCA**
**miR-1269a-F**	**GACTGAGCCGTGCTACTGG**
**miR-1269a-R**	**TGTCGTGGAGTCGGCAATTG**
**miR-105-F**	**GCCCTCGAGATACCATATCTATCCCCTTTTTCA**
**miR-105-R**	**GCCGAATTCCAACCATGAAGATACGAATTGATG**
**miR-522-3p-F**	**GGGCTCTAGAGGGAAGCGC**
**miR-522-3p-R**	**CAGTGCGTGTCGTGGAGT**
**miR-552-5p-F**	**CCGCACAGGTGACTGGTTAGA**
**miR-552-5p-R**	**GTGCAGGGTCCGAGGT**
**SFRP2-F**	**ACGGCATCGAATACCAGAACA**
**SFRP2-R**	**CTCGTCTAGGTCATCGAGGCA**
